# Exposed Pulps

**Published:** 1899-01

**Authors:** 


ARTICLE VI.

### EXPOSED PULPS.

Dr. Patterson: Pericemental inflammation, after pulp removal, can always be avoided by removing the pulp by surgical methods. Cocaine can be forced into the pulp until it is completely anæsthetized, using very finely pulverized crystals of cocaine. With the chloride of ethyl spray the pulp can be so congealed that cocaine can be forced into it by means of pressure with spunk, cotton or unvulcanized gutta-percha. The pulp can then be removed absolutely without pain.

Dr. Taft abandoned the use of arsenic twenty years ago. Its effects are objectionable, not only upon the pulp itself, but by extending to other tissues. The results are too uncertain. With the increase of facilities, other methods are so efficient that there is no longer any excuse for its use. Cocaine, finely pulverized and dissolved, is readily taken into the pulp by absorption, until it is so anæsthetized that it can be removed with as little pain as a paring from the finger-nail. When the pulp is removed by surgical procedure and all soft tissues removed from the canal and hemorrhage arrested, then is the time to close it. Seal it up immediately, and there is no liability of any changes pernicious to its welfare.

Dr. Watkins prefers, when he has a large cavity with free access, to punch it out with a slim piece of orange wood. It is instantaneous, the pulp is removed entire, and the tooth is ready for filling.

Dr. Barrett: Those who oppose arsenic as a dangerous drug, advise the use of cocaine! Which is the most dangerous? It is "jumping from the frying-pan into the fire."

Dr. H. B. Smith: The surgeon who uses cocaine knows the liabilities. He is prepared to combat evil effects, which he knows how to counteract. The trouble with arsenic is, that we do not know how far-reaching its effects may be in any case.

Dr. Crawford spoke of the importance of preventing septic infection (which he prefers to call toxic invasion) by stopping each root as soon as the canal is emptied, not leaving it open to serve as a means of self-destruction, while operating in the other canals.—*Ohio Journal.*

